# Research progress on the impact of intratumoral microbiota on the immune microenvironment of malignant tumors and its role in immunotherapy

**DOI:** 10.3389/fimmu.2024.1389446

**Published:** 2024-07-05

**Authors:** Jiamei Xu, Min Cheng, Jie Liu, Mengqi Cui, Beibei Yin, Jing Liang

**Affiliations:** ^1^ Department of Oncology, The First Affiliated Hospital of Shandong First Medical University & Shandong Provincial Qianfoshan Hospital, Shandong Key Laboratory of Rheumatic Disease and Translational Medicine, Shandong Lung Cancer Institute, Jinan, China; ^2^ School of Clinical Medicine, Weifang Medical University, Weifang, China

**Keywords:** intratumoral microbiota, tumor immune microenvironment, immunosuppressive microenvironment, immunotherapy, engineering bacteria

## Abstract

Microbiota has been closely related to human beings, whose role in tumor development has also been widely investigated. However, previous studies have mainly focused on the gut, oral, and/or skin microbiota. In recent years, the study of intratumoral microbiota has become a hot topic in tumor-concerning studies. Intratumoral microbiota plays an important role in the occurrence, development, and response to treatment of malignant tumors. In fact, increasing evidence has suggested that intratumoral microbiota is associated with malignant tumors in various ways, such as promoting the tumor development and affecting the efficacy of chemotherapy and immunotherapy. In this review, the impact of intratumoral microbiota on the immune microenvironment of malignant tumors has been analyzed, as well as its role in tumor immunotherapy, with the hope that it may contribute to the development of diagnostic tools and treatments for related tumors in the future.

## 1 Introduction

Intratumoral microbiota has been confirmed to exist, including bacteria, fungi, viruses, archaea and protist, which has been widley studied as an important part of tumor microenvironment (TME) with tumor specificity (1). There are three main sources of intratumoral microbiota: (1) Microbiota in the oral cavity, intestines, and other areas, which reaches the tumor site through blood circulation; (2) microbiota in the mucosa where the tumor is located, which enters the TME through the mucosal barrier; and (3) those derived from normal tissue adjacent to the tumor (1, 2). With the development of gene sequencing technology, intratumoral microbiota could be well detected, making it possible to study its impact on tumor occurrence, development, and treatment response (3). The tumor immunotherapy refers to enhancing the anti-tumor immune response of the body by using immunological methods to activate immune cells. Due to its specificity in attacking tumor cells and limited side effects, it has been becoming one of the most promising treatment methods (4). The FDA has approved the clinical application of CTLA-4 and PD-1/PD-L1 inhibitors, and other monoclonal antibodies, for the anti-tumor immunotherapy in patients with malignant tumors. The main mechanism underlying these drugs is to activate the T cells to kill tumor cells by blocking the inhibiting signal of T cell activation, which could achieve significant effects in the clinical treatment of melanoma and non-small cell lung cancer. However, the immune checkpoint therapy has no significant effect on tumors with poor T cell infiltration and low tumor antigen mutation (such as pancreatic cancer and ovarian cancer) (5). By comparing the survival time of patients with or without antibiotic treatment, Tucker and Rini et al. have found that immunotherapy was more effective for patients receiving no antibiotic treatment. These findings indicate that the intratumoral microbiota affects the tumor immunotherapy by regulating local or systemic anti-tumor immunity (6). The microbiota and its metabolites can affect the efficacy of immunotherapy by regulating the body’s immune system. Nejman et al. have found that among patients with Melanoma who respond to immune checkpoint inhibitors, *Clostridium* is more abundant in the tumor, while among those who do not respond, *Gardnerella vaginalis* is more abundant in the tumor (1). These findings indicate that the intratumoral microbiota promotes the efficacy of anti-tumor therapy by participating in the immune response.

## 2 Role of intratumoral microbiota in occurrence and development of malignant tumors

Studies have shown that there are a large number of microorganisms in tumor tissue, some of which may change the TME and participate in the occurrence and development of tumors. Intratumoral microbiota can promote tumor development through three pathways: (1) causing DNA damages with metabolites and secretions, leading to genomic instability; (2) participating in signal transduction in the signaling pathways; and (3) affecting the immune response (7). *Escherichia coli* could be divided into the A, B1, B2, and D groups, respectively. About 35% of the isolates in Group B2 have a 54kb pks island, which encodes a non-ribosomal peptide synthetase (NRPS)-polyketide synthase (PKS) hybrid gene cluster, encoding colibactin. Colibactin can cause DNA damages in mammals, thereby promoting genomic instability and inducing tumor development (8). In addition to causing DNA damages, intratumor bacteria also participate in cell signal transduction (9). *Helicobacter pylori* (Hp) produces cytotoxin-associated gene A(Cag A), which activates the β-catenin pathway, enters the cytoplasm of host cells and induces tumor (10). NF-κB is a transcription factor that promotes tumorigenesis (11). Dysbiosis of intratumoral microbiota can activate NF-κB, and the activation of NF-κB affects the immune response, thus promoting the occurrence of tumors (12).

**Figure 1:**
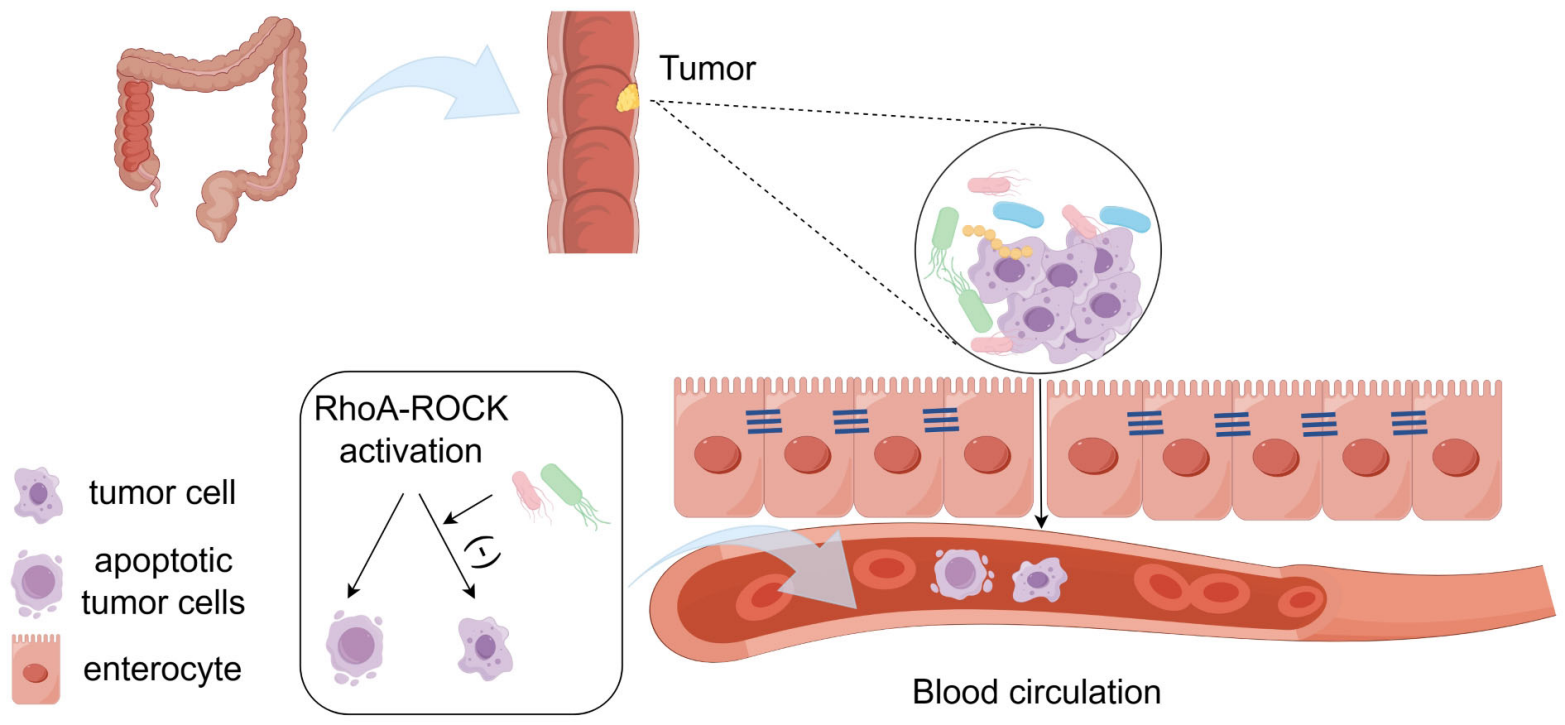
The intratumoral microbiota promotes tumor metastasis. Microbiota can break down the gut vascular barrier and promote the tumor metastasis. Microbiota inhibits the activation of RhoA and ROCK in cells and reduces the contractility, which is beneficial for the survival of circulating tumor cells under shear stress in the circulation. (-), it means inhibition.

In addition to promoting the occurrence of tumors, intratumoral microbiota can also promote the growth and metastasis of tumors. It has been shown that, some microorganisms can promote the proliferation of Treg cells and myeloid-derived suppressor cells(MDSCs), and promote the tumor growth (13). Ge Zhang et al. have shown that infection with *Fusobacterium nucleatum*(*F. nucleatum*) would stimulate tumor cells to release tumor-derived exosomes (TEXs), thereby promoting tumor cell metastasis in patients with colon cancer. Exosomes are extracellular vesicles secreted by cells, which can transmit proteins and genetic information between cells. TEX transfers miR-1246/92b-3p/27a-3p and CXCL16/RhoA/IL-8 from cells infected with *F. nucleatum* to uninfected cells, promoting tumor cell migration (14). Francesco Curcio et al. have shown that tumor cells can activate Toll-like receptor 4 (TLR4), which belongs to the pattern recognition receptors (PRRs) family. TLR4 can recognize pathogen-associated molecular patterns(PAMP) and damage-associated molecular patterns(DAMP). Some bacterial components would be PAMP, such as lipopolysaccharide (LPS), which is one of the main components of the cell wall of gram-negative bacteria (15). The expression of TLR4 is up-regulated by LPS in the cell wall of *E. coli* in tumors. After activation, TLR4 can induce inflammatory signals, promote tumor cell proliferation, and facilitate its escape from immune surveillance, thus promoting tumor metastasis (16). Majority of the malignant tumor patients’ death would be related to the tumor metastasis and spread. The key pathway for tumor metastasis is associated with the circulatory system, through which the cells would settle in suitable areas to proliferate. The involved factors mainly include the vascular endothelial integrity or microvascular permeability, hydrodynamic factors, microvascular geometry, cell adhesion molecules and surrounding extracellular matrix (17). Microbiota can break down the gut vascular barrier and promote the tumor cell metastasis (18). Tumor cells undergo fluid shear stress after injected into the circulatory system, which may lead to cellular apoptosis (19). The intratumoral microbiota can inhibit the activation of Actin Cytoskeleton tissue (RhoA and ROCK) in cells and reduce the contractility. The reduction of contractile force induced by bacteria is beneficial for the survival of circulating tumor cells under shear stress in the circulation, thereby enhancing the colonization of tumor cells in other sites (20). (**Figure 1**) Some microorganisms would promote the recruitment and activation of CD8 T cells, increase IFN-γ, TNF-α and IL-2 in serum, and promote the anti-tumor immune response (13). Compared to drugs, bacteria have motility, which can reach and proliferate at any part of tumor tissue. Most bacteria compete with tumor cells for the necessary nutrients for survival. The presence of bacteria can also activate the body’s immune system, indirectly leading to tumor cell death (21). Based on the above situation, the intratumoral microbiota plays an important role in the occurrence, development, and metastasis of tumors (**Table 1**). Therefore, studying the impact of intratumoral microbiota on the TME and immunotherapy is of great significance.

**Table 1:** The features of intratumoral microbiota in different tumors.

Cancer	Dominant intratumoral microbiota	Potential function	Refs
Liver cancer	*Akkermansia*	Contribute to a good prognosis	(22)
	*Muribaculaceae bacterium isolate 102 HZI, Ralstonia sp UNC404CL21Col*, and *Allobacillus sp SKP48*	Regulate the tumor microenvironment	(23)
	*Pseudomonadaceae*	Have antitumor effect	(24)
Breast cancer	*Firmicutes*	Promote metastasis and colonization	(20)
	*Fusobacterium nucleatum*	Promote metastasis	(25)
	*Enterotoxigenic Bacteroides fragilis*	Promote tumor progression	(26)
Pancreatic Cancer	*Proteobacteria, Bacteroidetes, Firmicutes, Actinobacteria, Pseudomonas*, and *Elizabethkingia*	Promote tumor progression	(27)
	*Acinetobacter, Pseudomonas*, and *Sphingopyxis*	Promote tumorigenesis	(28)
	*Pseudoxanthomonas, Streptomyces, Saccharopolyspora*, and *Bacillus clausii*	Contribute to the anti-tumor immune response	(29)
	*Gammaproteobacteria*	Induce gemcitabine resistance	(30)
Colorectal cancer	*Fusobacterium nucleatum, Bacteroides fragilis, Bacteroides thetaiotaomicron*, and *Prevotella intermedia*	Promote tumor growth and metastasis	(31)
	*Fusobacterium, Bacteroides, Parvimonas*, and *Prevotella*	Promote tumorigenesis	(32)
	*Fusobacterium nucleatum* and *Bifidobacterium*	Promote metastasis	(33)
Lung cancer	*A. sydowii*	Promote tumor progression	(34)
	*Roseburia*	Affect tumor recurrence	(35)
	*Koribacteraceae* and *Lachnospiraceae*	Affect survival	(36)

## 3 Impact of intratumoral microbiota on TME and immunotherapy

### 3.1 Hepatocellular carcinoma

**Figure 2:**
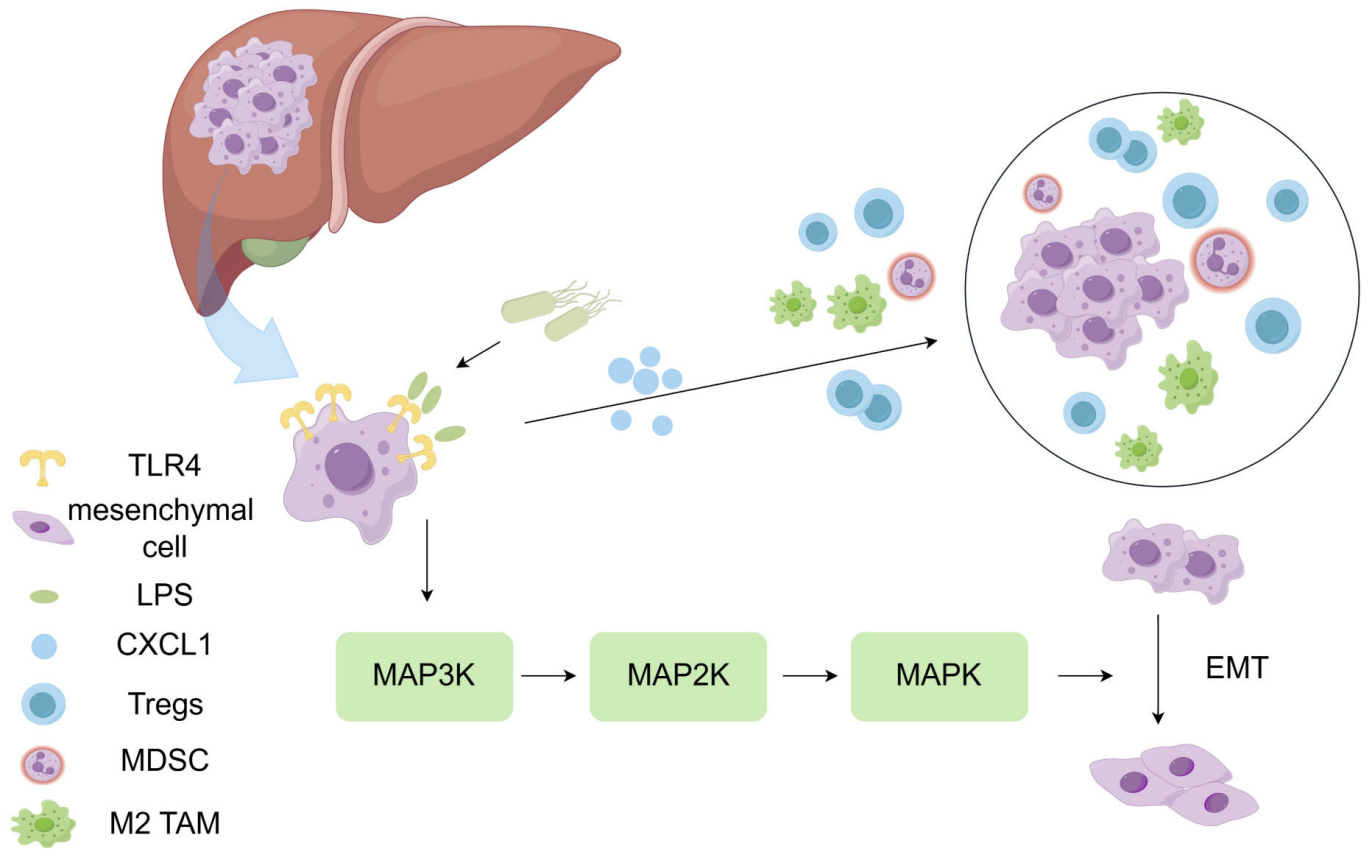
LPS contributes to the formation of immunosuppressive microenvironment. TLR4 can recognize LPS in the cell wall of Gram-negative bacteria, stimulate CXCL1 expression in hepatocytes, activate the JNK/MAPK signal transduction, and promote the epithelial-mesenchymal transition (EMT) of HCC cells. Overexpression of TLR4 in hepatocellular carcinoma contributes to the formation of immunosuppressive microenvironment.

The presence of intratumoral microbiota in liver has been confirmed. It has been found that the most abundant microbial species in the liver microenvironment include the *Patesibacteria, Proteobacteria*, *Bacteroidetes*, *Firmicutes*, and *Actinobacteriota (* 37). The carcinogenic or inhibitory effects of intratumoral microbiota depend on their regulation of the immune system. On one hand, microbiota can lead to chronic inflammation and promote tumor development. Moreover, it can lead to immune activation, which in turn eliminates tumor cells (38). Overexpression of TLR4 in hepatocellular carcinoma contributes to the formation of immunosuppressive microenvironment. Specifically, TLR4 can recognize LPS in the cell wall of Gram-negative bacteria, stimulate the expression of CXCL1 in hepatocytes, activate the JNK/MAPK signal transduction, and promote the epithelial-mesenchymal transition (EMT) of HCC cells, which is conducive to the formation of immunosuppressive TME (39). (**Figure 2**) Qu et al. have found that there is no significant difference in the microbial diversity between the tumor tissue and the adjacent non-tumor tissue. However, at the family or genus level, due to the anti-tumor effect, *Pseudomonas* is significantly decreased in tumor tissue, while *Rhizobiaceae* and *Agrobacterium* are significantly increased in tumor tissue. Patients with relatively high abundance of *Pseudomonas* have better prognosis (24). On the contrary, Huang et al. have found that the α diversity of microbiota in HCC is significantly higher than the normal liver. The α diversity (single sample diversity analysis) is used to describe the diversity and abundance of bacterial species in a single sample (37, 40). There may be several reasons for the inconsistent findings from these studies: (1) The non-tumor tissue selected in the experiment are affected by the diseased liver tissue; (2) all the samples are taken from patients with malignant tumor, and there is no contrast between normal liver tissue; (3) it may be contaminated during DNA extraction, PCR amplification and/or sequencing; and (4) the sample size is insufficient. In addition to affecting immune response, intratumoral microbiota can also enhance the biosynthesis of fatty acids and lipids, thus promoting the proliferation of tumor cells (40). Combined immunotherapy based on immune checkpoint inhibitors has become the first-line treatment for advanced hepatocellular carcinoma. Considering that the intratumoral microbiota can regulate the body’s immune response, targeting the microbiota to enhance the effects of immunotherapy is worth further study.

### 3.2 Breast cancer

Nejman et al. have found that the breast tumor tissue is rich in *Staphylococcus*, *Lactobacillus*, *Enterococcus*, and *Streptococcus*. Compared with the lung cancer, ovarian cancer, pancreatic cancer, melanoma, bone tumor and brain tumor, the diversity of microbiota in the breast cancer is increased (1). Fu et al. have used a mouse spontaneous breast tumor model, i.e., the mouse mammary tumor virus-polyoma middle T-antigen (MMTV-PyMT), which is similar to human breast cancer, to study the intratumoral microbiota. They have found that the bacteria in tumor tissue are similar to those in human beings, and the intracellular bacteria can change the activity of host cells and promote tumor progress (20). *F. nucleatum* can specifically bind to the abundant Gal-GalNAc on tumor cells of patients with breast cancer through the lectin Fap2 on its surface, so as to colonize breast tumors. *F. nucleatum* can accelerate the progress of breast cancer and tumor metastasis by inhibiting the aggregation of CD4 T cells and CD8 T cells in TME after colonization. Moreover, *F. nucleatum* can inhibit the anti-tumor immunity by activating two human immunesuppression checkpoint receptors (the TIGIT and CEACAM1) (25). The abundance of *Yanoikuyae* is sharply decreased in breast tumor tissue compared with normal breast tissue, indicating that this microorganism may play an anti-tumor role in the breast (41). *Yanoikuyae* expresses glycosphingolipid ligands, and then activates invariant natural killer T (iNKT) cells. The iNKT cells are CD1d-restricted T lymphocytes, which can recognize specific lipid antigens. The iNKT cells represent important medium for tumor immune monitoring which play important roles in inhibiting the growth and metastasis of breast tumors (42).

### 3.3 Pancreatic cancer

With the development of sequencing technology and increasing investigation on pancreatic cancer, the tumor microenvironment, which has previously been considered absolutely sterile, has been found to contain microorganisms (43). It has been found that the α diversity of intratumoral microbiota in long-term survivors of pancreatic cancer is higher than that of short-term survivors, and long-term survivors have characteristic microorganisms (29). Pushalkar et al. have found that *Proteobacteria phylum*, *Bacteroides phylum*, and *Firmicutes phylum* are the most abundant and prevalent bacterial groups in pancreatic cancer. The microbiota can induce the peritumor immunosuppression and promote the progression of pancreatic cancer. Targeting the microbiota would prevent the tumorigenesis and reverse the immune tolerance of tumor cells, thus making the checkpoint-based immunotherapy effective (27). The microbiota can generate tolerant immune response through differential activation of selected Toll-like receptors (TLRs) in monocyte. Ablation of the intratumoral microbiota can inhibit the infiltration of MDSCs, enhance the M1 macrophage differentiation, and promote the activation of CD4 T cells and CD8 T cells. It can also up-regulate the TNF-α and IFN-γ (which have anti-tumor effects), inhibiting the progression of malignant tumors (44, 45). In addition to bacteria, tumor tissue in pancreatic cancer is also rich in fungi. Berk Aykut et al. have found that fungi in gut can migrate to pancreas (46). Compared with normal pancreatic tissue, patients with pancreatic cancer have higher abundance of fungal communities in tumors, the most abundant of which are *Malassezia* and *Alternaria (* 47, 48). IL-33 is a pro-inflammatory cytokine released by cancer cells. It can combine with the cognate receptor ST2 to recruit Th2 and ILC2 cells. Th2 and ILC2 cells secrete IL-4, IL-5 and IL-13 to promote tumor development. The fungi in the tumor can promote the secretion of IL33. After treatment with antifungal drugs, the fungi in the tumor would be decreased, leading to obvious regression of the tumor, and the infiltration of Th2 and ILC2 would also be decreased (48). Dominant microorganisms within tumors, such as *Saccharopolyspora*, *Pseudoxanthomonas*, and *Streptomyces*, are beneficial to promote the recruitment and activation of CD8 T cells, which can produce plenty of anti-tumor cytokines (such as TNF-α and IFN-γ), thereby enhancing the anti-tumor immune response (29). The intratumoral microbiota in patients with pancreatic cancer would promote the production of the IL-1β. IL-1β derived from tumor cells can activate pancreatic stellate cells (PSCs), which in turn leads to mesenchymal fibrosis, inhibiting T cell infiltration (49, 50). The *Megasphaera* specifically enriches and produces plenty of short-chain fatty acids in long-term survivors, which promotes the production of effector cytokines and enhances the immune response (51). The immune tolerance may lead to poor effects of immunotherapy drugs on pancreatic ductal adenocarcinoma (PDAC). Therefore, it has a positive impact on the treatment of pancreatic cancer by regulating immunotherapy through microbiota.

### 3.4 Colorectal cancer

There are a large number of microorganisms in human intestine. The dysbiosis of gut microbiota is involved in the occurrence and development of intestinal tumors. *Bacteroides fragilis* (*B. fragilis*) has been commonly seen in human intestine. *Bacteroides fragilis* can be divided into two groups according to the ability to secrete *Bacteroides fragilis* toxin (BFT), i.e., the enterotoxigenic *B. fragilis* (ETBF) and nontoxigenic *B. fragilis* (NTBF) (52). Studies have confirmed that *B. fragilis* is more common in cancer patients than normal people, mainly ETBF. ETBF can promote chronic inflammation and drive the occurrence of colorectal cancer (53). It has been found that the dominant phyla in tumor tissue of colorectal cancer patients are *Firmicutes phylum* and *Bacteroides phylum*, and the secondary dominant phyla include *Proteobacteria*, *Fusobacteria*, and *Actinobacteriota (* 54). *Escherichia coli* in colorectal cancer patients can migrate to the liver when the intestinal vascular barrier is disrupted, which would then activate the liver microenvironment by recruiting innate immune cells, thus promoting tumor metastasis (55). There are abundant *F. nucleatum* in the colorectal cancer. Bullman et al. have reported that in the patient-derived tumor xenograft (PDX) model containing *Fusobacterium*, treatment of antibiotics would reduce the load of *Fusobacterium* in tumor and inhibit the proliferation and growth of tumor cells. Based on these, we infer that *F. nucleatum* can affect the immune response, thus affecting the efficacy of immunotherapy (31). Microbiota can enhance T cell response in different tumors and improve the therapeutic effect of the anti-PD-L1 therapy (56). Jung Ho Kim et al. have found that among colorectal cancer patients with high microsatellite instability, patients with high content of intratumor *F. nucleatum* have larger tumors and are more prone to suffer from late invasion beyond the intrinsic muscle layer, compared with those with low content (without *F. nucleatum*) *(* 57). However, the mechanism of the tumor immune interaction between intratumor *F. nucleatum* and colorectal cancer has not been fully elucidated, and further investigation is still needed. If this mechanism is clarified, *F. nucleatum* in TME is expected to become one of the targets of immunotherapy.

### 3.5 Lung cancer

In normal lungs, the most abundant microorganisms include *Firmicutes*, *Actinobacteriota*, *Bacteroidetes*, and *Proteobacteria*, and the core microorganisms include *Veillonella*, *Haemophilus*, *Neisseria*, *Streptococcus*, *Fusobacterium*, and *Prevotella (* 58). Lee et al. have found that the microorganisms significantly enriched in lung cancers are *TM7* and *Firmicutes* at the phylum level, and *Megasphaera*, *Selenomonas*, *Atopobium*, and *Veillonella* at the genus level (59). In 2020, Lindsay et al. have grouped patients with lung adenocarcinoma (LUAD) and lung squamous cell carcinoma (LUSC) based on age and sex, and then they have studied whether there are differences in the composition of microbiota in each cohort. In LUSC, *Pseudomonas putida* str. KT2440 is common in young male patients, which can participate in immune regulation. Specifically, patients with higher abundance of *Pseudomonas putida* str. KT2440 have more neutrophils and monocytes, while CD8 T cells and macrophages are fewer. In LUAD, *Escherichia coli* str. K-12 substr. W3110 is related to the survival rate and genome changes of elderly females and males (60). The intratumoral microbiota of lung cancer can produce TLR ligands (such as lipopolysaccharide and peptidoglycan), stimulate myeloid cells to produce Myd88-dependent IL-1b and IL-23, induce the proliferation and activation of gamma delta (γδ) T cells, and then produce other pro-inflammatory cytokines, such as IL-17, which can promote the occurrence of inflammation and the growth of tumor cells (61).

Intratumoral microbiota not only participates in the immunomodulation of tumors mentioned above, but also plays a role in the immunotherapy of other tumors. Mackenzie J. Bender et al. have found that *Lactobacillus reuteri* can colonize melanoma continuously. When mice are fed on food rich in tryptophan, *Lactobacillus reuteri* can release indole-3-aldehyde (I3A), which can activate the aryl hydrocarbon receptor (AhR) within CD8 T cells, and enhance the therapeutic effect of immune checkpoint inhibitors (62). *Bifidobacterium* derived from the gastrointestinal tract can accumulate within tumors and enhance immunotherapy efficacy through STING signaling (63). To sum up, the intratumoral microbiota can regulate the immune response and affect the efficacy of immunotherapy.

## 4 Engineering bacteria

Engineering bacteria refer to the microorganisms that are modified based on natural bacteria using modern biotechnology to deliver drugs, induce apoptosis of tumor cells, and cause anti-tumor immunity. Engineering bacteria can target tumors and accumulate in TME. Microorganisms have better permeability in tumor tissue than drugs, which can reach the depth of tumor tissue and kill tumor cells comprehensively (21, 64). In the absence of external force, some anaerobic or facultative anaerobic bacteria can invade or colonize solid tumors, and enter the tumors that cannot be completely penetrated by passive treatment, thereby inhibiting the tumor growth or eliminating the tumors. Some bacteria are toxic and can cause immune response in the body. After entering the solid tumor, the engineering bacteria are less susceptible to be attacked by the body’s immune system, and therefore can continue to release the anti-tumor drugs and inhibit the growth of tumor cells (65). Due to the heterogeneity of intratumoral microbiota, engineering bacteria can avoid killing healthy tissue and reduce the side effects compared with other drugs. Engineering bacteria can also alter the tumor microenvironment and enhance the host’s anti-tumor immune response by regulating immune response and/or metabolism. With the development of modern biotechnology, many engineering bacteria have been studied for the treatment of tumors. Attenuated *Salmonella typhimurium* has the characteristic of selective growth in tumors. Yoon et al. have genetically modified the bacterium to express IFN-γ, which has a greater cytotoxic effect and can inhibit tumor growth and prolong survival in mouse model (66). Geng et al. have bound aptamer to the surface of bacteria, and compared with unmodified bacteria, aptamer conjugated *Salmonella* has a stronger ability to activate tumor immune response and exert better anti-tumor efficacy (67). Sreyan Chowdhury et al. have designed a non-pathogenic *Escherichia coli* strain, which can colonize tumors and locally release an encoded nanobody antagonist of CD47, increasing the activation of tumor-infiltrating T cells, and generating anti-tumor immunity (68). Fernando P Canale et al. have developed an engineering probiotic *Escherichia coli* Nissle 1917 strain, which can colonize tumor and convert ammonia into L-arginine. The increase of L-arginine concentration in the tumor can increase the number of tumor-infiltrating T cells, thus improving the efficacy of immunotherapy. In addition, this strain can synergistically enhance the therapeutic effect of PD-L1 blockade (69, 70).

## 5 Conclusion and outlook

At present, the researches only characterize the intratumoral microbiota and their effects on the occurrence, development and treatment of tumors in some tumors. We made a table to summarize clinical trials related to microbiota (**Table 2**). In the future, the researchers need to study more types of tumors and more researches are needed to elucidate the mechanism by which intratumoral microbiota affects the efficacy of treatment, particularly the relationship between intratumoral microbiota and immune response, as this may be a key factor in regulating the efficacy of immunotherapy. In addition, it is of great significance to balance the relationship between the therapeutic effect and the inflammation caused by microbiota. Some of the intratumoral microbiota has been used in the clinical treatment without safety assessment. It is necessary to conduct more clinical researches in the future. And the composition of intratumoral microbiota in some tumors has not yet been determined. In the future, more advanced and accurate technology is needed to be invented and used for microbiota sequencing, so as to find and develop new treatments for malignant tumors by using the intratumoral microbiota.

**Table 2:** Clinical trials related to intratumoral microbiota.

Tumor	Clinical Trial ID	Primary purpose	Status
Gastric adenocarcinoma	NCT05800236	To define whether intratumoral bacteria exist in gastric cancer	Recruiting
Triple Negative Breast Cancer	NCT03586297	To correlate gut and intratumoral microbiome composition and anti-tumor immune response with pCR	Recruiting
Colon Cancer	NCT04312360	To investigate the effect of local antibiotic treatment with fosfomycin and metronidazole on tumor characteristics and the colonic biofilm in patients with right-sided colon cancer or right-sided colon adenomas	Completed
Epidermoid Carcinoma of the Upper Aerodigestive Tract	NCT06061705	To evaluate the evolution of the presence of the bacterium in saliva, as well as the specific immune response to F. nucleatum in patients with CEVADS during immunotherapy treatment	Not yet recruiting
Ovarian Cancer	NCT06272240	To generate ovarian cancer organoids to characterize *in vitro* interactions and molecular pathway among tumor cells, immune cells, and resident microbiota (intratumoral bacteria and/or microbial-derived molecules)	Recruiting

In this paper, the role of intratumoral microbiota in the occurrence and development of tumors and their influence on the immune microenvironment of five different tumors and the immunotherapy of malignant tumors have been reviewed. Intratumoral microbiota and their products in tumors have both tumor-promoting and -inhibiting effects, depending on different cases and situations. Intratumoral microbiota can affect the immune response and serve as an emerging biomarker in immunotherapy. Antibiotic therapy that precisely targets on the intratumoral microbiota seems to be a promising treatment option, but its clinical safety and efficacy are still needed to be evaluated. The mechanisms of immune response and immunotherapy between the intratumoral microbiota and the host are still not fully understood, and more relevant researches are still needed in the future to gain in-depth understanding. Exploring changes in the composition of intratumoral microbiota to regulate its impact on immune status can help to develop new treatment methods for cancers in clinic.

## Author contributions

JX: Writing – original draft. MCh: Writing – original draft. JLiu: Writing – original draft. MCu: Writing – original draft. BY: Writing – review & editing. JLia: Writing – review & editing.
